# Archaeological evidence for long-term human impacts on sea turtle foraging behaviour

**DOI:** 10.1098/rsos.240120

**Published:** 2024-07-17

**Authors:** Eric Guiry, J. Ryan Kennedy, Corey Malcom, Mariah Miller, Olivia Hall, Michael Buckley, Paul Szpak

**Affiliations:** ^1^ Department of Anthropology, Trent University, 1600 West Bank Drive, Peterborough, Ontario K9L 0G2, Canada; ^2^ School of Archaeology and Ancient History, University of Leicester, Mayor’s Walk, Leicester LE1 7RH, UK; ^3^ Department of Anthropology, Indiana University Bloomington, 701 East Kirkwood Avenue, Bloomington, IN 47405, USA; ^4^ Florida Keys History Center, 700 Fleming Street, Key West, FL 33040, USA; ^5^ School of Natural Sciences, Manchester Institute of Biotechnology, University of Manchester, 131 Princess Street, Manchester M17 DN, UK

**Keywords:** sea turtles, conservation, stable isotopes, historical ecology, seagrasses, Caribbean sea

## Abstract

Early conservation efforts to prevent the loss of green sea turtles (*Chelonia mydas*) from the Caribbean Sea jumpstarted marine habitat and biodiversity protection. However, even there, limitations on historical observations of turtle ecology have hampered efforts to contextualize foraging behaviours for conservation management. We integrate isotopic and zooarchaeological evidence from green sea turtles harvested at the Miskito Cays (Nicaragua) to assess foraging behaviour before and after a step change in harvesting intensity. Highly structured isotopic evidence shows greater foraging adaptability in earlier populations. This provides a counterpoint to recent synthesis, suggesting the ecological non-exchangeability of sea turtles, which complicates conservation planning focused on genetic-stock-based repopulation. In contrast, our results suggest future populations would have a capacity for higher degrees of ecological exchangeability than current perspectives allow. This highlights a need to consider the kinds of longer term perspectives, such as those offered by archaeological materials, when planning for future sea turtle recovery.

## 1. Introduction

There has been a global explosion of publications of stable isotope research on sea turtles in recent years [1–4], much of which has been oriented towards understanding fundamental aspects of their ecology and helping to contextualize and facilitate conservation planning. Despite this dramatic increase in activity, it is still rare for isotopic approaches to incorporate retrospectives using historical, archaeological or palaeontological sample materials. Given the long history of human exploitation of sea turtles, especially during and after the rise of industrial-scale harvesting in various regions over the last few hundred years, these longer term perspectives can provide essential context for how sea turtles might behave in a future where their population sizes have recovered to levels from before unsustainable harvests began. To the extent that conservation work is (or could be) aimed at realizing this kind of idealized future, it is reasonable to expect that the incorporation of archaeological materials (which, globally, represent a vast biomolecular archive of samples spanning millennia) will have a major role to play in developing more robust conservation management strategies.

**Figure 1.**
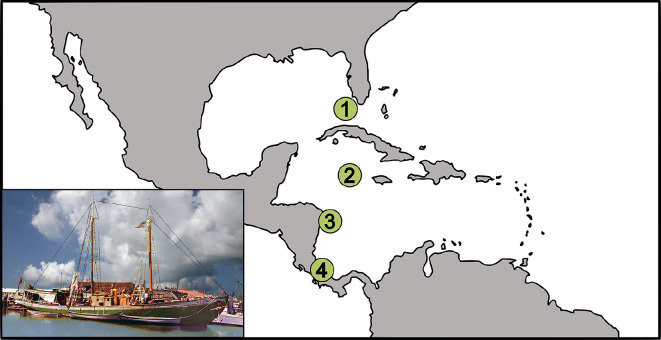
Map showing locations of key sites in the context of the Caribbean Sea and Gulf of Mexico: (1) Key West Turtle Kraals and Cannery (USA); (2) the Cayman Islands; (3) the Miskito Cays seagrass pastures and turtling grounds (within Nicaragua’s Región Autónomo del Atlántico Norte); (4) Tortuguero nesting beaches (Costa Rica). Inset shows the turtle schooner *A. M. Adams* in Key West Bight, *ca* 1960. Courtesy of the Florida Keys History Center/Monroe County Public Library.

In this study, we use isotopic analyses of a large sample of archaeological green sea turtle (*Chelonia mydas*; dating to the 1950s and 1960s) bones to identify changes in the species’ behaviour around the Miskito Cays (figure 1) before and after (using published comparative data from the 2010s) a step change in turtle harvesting driven by local industrialization of turtle processing in the region around 1970. By contextualizing these data within frameworks from seagrass ecosystem isotope ecology, different pools of traditional ecological knowledge (TEK) and narratives of the history of sea turtle conservation, we demonstrate how green sea turtle foraging behaviour has previously been more adaptable than has been observed in recent isotopic research. These changes in the apparent flexibility of green sea turtle foraging strategies have implications for conservation planning and highlight the value of longer term perspectives, such as those offered by archaeological materials, when planning for sea turtle recovery.

## 2. Context

Our analyses focus on the remains of green sea turtles that were harvested in the mid-twentieth century (*ca* 1950–1970), most likely from around the Miskito Cays (encompassing offshore bars, banks and cays off the coasts of Nicaragua and Honduras; see §4 for evaluation of harvesting location), and which were later recovered from rescue excavations of archaeological deposits from the Key West Turtle Kraals in the USA (figure 1) [5]. These remains represent large juvenile or adult-sized turtles that had been drawn into a complex harvest, processing and distribution system at the heart of a large international turtle soup industry. Within a framework of regional history and isotopic research, these specimens provide an ideal context for demonstrating the tremendous potential for isotopic retrospectives to drive forward intersecting aims of archaeological and ecological research on conservation-sensitive species.

The green sea turtle population in the Caribbean Sea played a critical role in developing conservation movements [6] and was the principle driver of Archie Carr’s seminal works [7–10] that initiated a wave of public concern and, ultimately, protection for marine ecosystems [11]. Carr’s work was spurred by declining green sea turtle populations, which had been witnessed across the Caribbean over centuries. Sea turtles had long been a critical resource for Indigenous and settler populations around the Caribbean, which led to their disappearance from areas in which they had once been extremely abundant [12]. This decline first became marked in the context of exploitation associated with historical European colonial projects, which saw turtles largely lost from places, such as the Florida Keys and the Cayman Islands [7,13–16], that had previously had such an abundance of access to these animals that turtles were considered an important pull factor in their initial settlement [7,17–21]. By the time Carr was writing in the 1950s, the turtle soup industry was the main driver of North American commercial turtling endeavours which had, already for decades, been shifting further and further afield to collect turtles as nearer waters were successively fished out [5,15,22–25].

**Figure 2.**
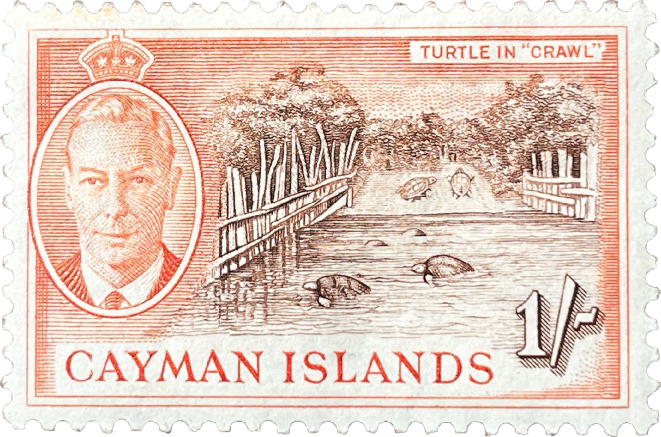
Government-issued stamp (*ca* 1950), highlighting the centrality of turtling to mid-twentieth century Caymanian identity. Scene depicts kraaling of green sea turtles long after Cayman Island nesting populations were extirpated. Scenes like this would still have been common at the time, with turtles caught and imported by Caymanian turtlers from distant places such as the Miskito Cays.

**Figure 3.**
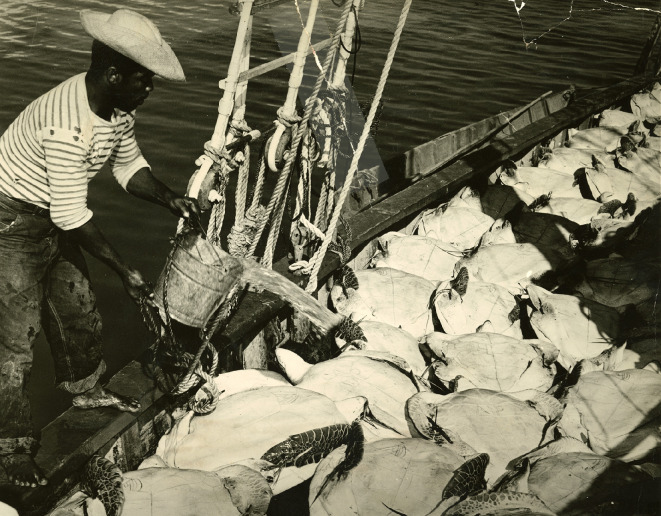
Crew member cooling green sea turtles with water on the deck of the *Adams* during transport in March 1956. Photo by the Florida State Advertising Commission. Scott De Wolfe Collection at the Florida Keys History Center/Monroe County Public Library.

The Key West Turtle Kraals were a major player in this saga and, by the early twentieth century, relied almost entirely on Grand Caymanian (hereafter, simply Caymanian) turtlers who brought with them generations of deeply rooted TEK on techniques for green sea turtle harvesting. Sea turtle harvesting, trade and consumption had become embedded in Caymanian national identity and culture (figure 2). After depleting local sea turtle populations by the nineteenth century, Caymanian turtlers began orchestrating a complex harvesting operation, involving long-distance voyages to the Miskito Cays [5,24,26,27]. Throughout the early to mid-twentieth century, sailing schooners like the *Adams*, a large purpose-built turtling vessel funded by owners of the Key West Turtle Kraals (figures 1 and 3), Caymanian turtlers would travel from Key West to the seagrass meadows around the Miskito Cays [5,21,24,26,27] (figure 1). There they would set nets at locations targeted to ensnare green sea turtles as they emerged from their nighttime sleeping areas. These turtles were considered adults by their harvesters (though may have included large juveniles), including both males and females [28,29], and typically weighed around 90 kg (ranging from 54.4 kg to 226.8 kg) [5,21]. Over the course of several weeks, Caymanian turtlers would aim to collect a full load of around 400 individuals before returning to Key West [5,26,27,30].

The green sea turtle’s namesake comes from the colour of their fatty and cartilaginous tissues (calipash and calipee), which is the main ingredient in turtle soup, and for soup production and canning operations to be successful, a large supply of healthy live turtles was needed at the Key West Turtle Kraals. In the context of Caymanian-led harvesting that sought to meet this demand for delivery of living, healthy turtles, the biological needs (caloric, thermal and respiratory) of green sea turtles required an intricate process of loading and unloading them from watercraft to kraals^1^ (figures 2 and 3), and from the turtle’s perspective, resulted in a protracted series of difficult situations.

**Figure 4.**
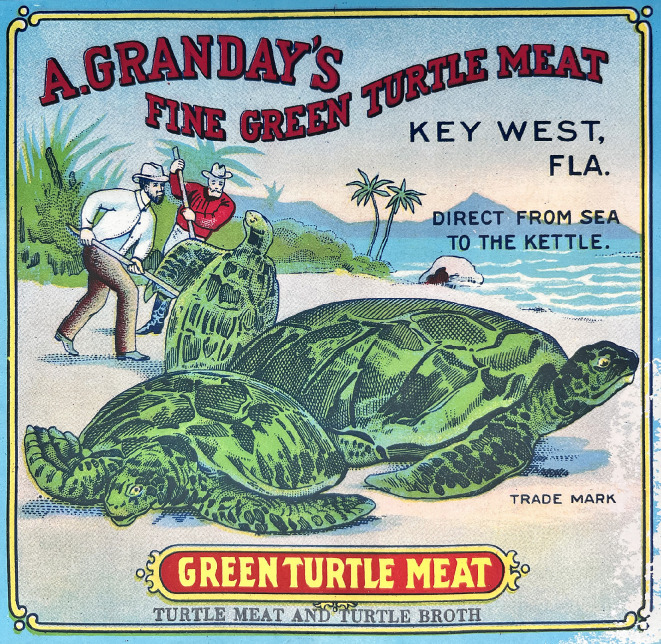
Consumer label from a green sea turtle product (*ca* 1890–1920) canned by Thompson Enterprises at the Key West cannery and sold in New York. Variants of this A. Granday brand label appeared on canned green turtle soup and meat products until the mid-twentieth century.

By the turn of the twentieth century, Key West was the nexus of America’s turtle soup industry, with several kraals, turtle purveyors and canneries [15,21,31,32]. Our samples come from the last of these to remain open, operated by Thompson Enterprises, which had consolidated the market and held a monopoly on green sea turtle products [5] (figure 4). Following a series of deterrents, including sea turtle conservation legislation and declining inter-generational transfer of Caymanian turtle harvesting TEK, the Key West Turtle Kraals ended importation and slaughtering operations in 1971 [5,27,28]. Shortly thereafter green sea turtles were formally listed as threatened and endangered in the USA’s Endangered Species Acts of 1973 as amended in 1978 [33].

The area surrounding the Miskito Cays plays a critical role in supporting a substantial portion of the world’s largest breeding population of green sea turtles [34,35]. The region’s coastal shelf provides habitat for immense seagrass meadows, dominated by turtle grass (*Thalassia testudinum*), which are heavily used as pasture for green sea turtles in the Caribbean Sea [35,36]. For these reasons, green sea turtles foraging in this area have received considerable attention from scientists interested in understanding their ecology with the aim of improving conservation outcomes (e.g. [6–8,9,10,21,29]). While this population had long been harvested by international communities, particularly Caymanians on behalf of American and other international consumers, it has also provided sustenance and a cultural focal point for local Indigenous communities, particularly the Miskito peoples, who still practice a substantial green sea turtle harvest today [37–41]. Using carcasses from this harvest, as well as tissues from live catch and release, researchers have been able to study how green sea turtles use the region’s seagrass pastures. A large stomach content study conducted on green sea turtles harvested in 1975 and 1976 confirmed both the herbivorous and seagrass focus of this population [36], but noted that, to varying degrees, marine algae, sponges and terrestrial flotsam were consumed as well. From a conservation perspective, however, these data have less utility because stomach content analyses, which only offer evidence for recently consumed foods, do not provide the kind of information on longer term dietary patterns needed to understand how the behaviours linking green sea turtles and seagrass could render them vulnerable to various threats.

Isotopic analyses of these green sea turtle populations have helped to further contextualize findings from stomach content studies. Compared with other marine primary producers targeted by green sea turtles, seagrasses typically have higher stable carbon (*δ* ^13^C) and lower stable nitrogen (*δ* ^15^N) isotope compositions making it possible to assess the presence of seagrasses in marine grazers’ diets (see §4). Stable carbon and nitrogen isotope composition of skin samples from 183 green sea turtles sampled from the same region between 2009 and 2011 provided short-term (compared with bone) perspectives on diet and indicated the importance of seagrass as well, but these data were not considered distinctive enough to quantify the relative importance of seagrass versus other foods [42]. Stable nitrogen isotope compositions from these samples, including both whole skin and its constituent amino acids, indicate that variation in green sea turtle *δ* ^15^N results from baseline rather than diet (omnivory) shifts in the kinds of foods and locations in which turtles were feeding [42]. This confirmed that green sea turtles in the wider Caribbean Sea are strictly herbivorous. Further serial-sampling analyses of shell scutes, which preserve a diachronic record for diet changes (because they form incrementally and do not remodel once laid down), from 21 adult green sea turtles, also collected in 2009 from a nearby nesting beach, indicated a surprising degree of inter-individual variability in terms of the *δ* ^13^C and *δ* ^15^N composition of foods being eaten, but very little intra-individual variation [43]. Again, however, without being able to link these data to the relative importance of seagrass, results were not sufficient to investigate links with specific habitat types aside from concluding that individual green sea turtles appear to specialize in particular, isotopically distinct foods and/or areas which include seagrass beds.

In the context of turtle behaviour, stable sulfur isotope (*δ* ^34^S) compositions have the potential to resolve the relative role of seagrass in green sea turtle diets. While primary producers in marine environments have access to a large pool of isotopically homogenous sulfate (*ca* +20‰ [44]), it is well documented that seagrasses, which are typically rooted in anoxic sediments, use sulfur that has been passed through a reduction (to sulfide) and reoxidation cycle and, therefore, will typically have systematically low *δ* ^34^S values [45–48] (see §4). Furthermore, these lower *δ* ^34^S values are most pronounced closer to the base of the plant [47]. In that context, the grazing behaviour of green sea turtles, which selectively crop and discard older materials, thus, consuming younger growth near the base of seagrasses [23,49–52], means that individuals that focus their diets on seagrasses are systematically consuming foods with the lowest and, in the context of other major marine foods (algae), most distinctive *δ* ^34^S compositions. Therefore, within existing isotopic frameworks for exploring green sea turtle diets, *δ* ^34^S offers an opportunity for better resolving variation in *δ* ^13^C and *δ* ^15^N by the relative importance of seagrass.

The biology of sea turtle bone, which grows outwards in concentric layers [53–55], adds another dimension of utility to archaeological remains for exploring past green sea turtle behaviour. Because these growth ‘rings’ are thought to undergo limited remodelling once laid down, their isotopic compositions can preserve a diachronic record of changing turtle diet and habitat use [56–59]. Like scutes, they can be incrementally sampled to explore diet through time at the intra-individual level [56]. This allows us not only to investigate and compare diachronic variation in long-term behaviour within and among individuals, but also to cross-reference and better calibrate the theoretical isotopic frameworks we use to interpret isotopic variation observed in whole-bone data. Together these advantages offer a valuable opportunity to deepen our knowledge of sea turtles’ isotope ecology more broadly.

In this study, we perform *δ* ^34^S, *δ* ^13^C and *δ* ^15^N analyses of whole-bone cross sections of 122 archaeological green sea turtle right humeri and additional serial sampling analysis on 12 of these individuals. The primary aim of this study was to understand both broad-scale inter-individual and comparatively fine-grained intra-individual variation in seagrass use among mid-twentieth-century turtles likely harvested around the Miskito Cays (see §4 for evaluation of harvesting location). In this context, analyses aimed to address three research questions: (i) to what extent can a seagrass-intensive diet impact marine consumers’ *δ* ^34^S? (ii) How variable are historical sea turtle diets and how do these compare with more recent populations? (iii) To what degree do individual turtles shift diet specializations over the last years of life?

## 3. Results

**Figure 5.**
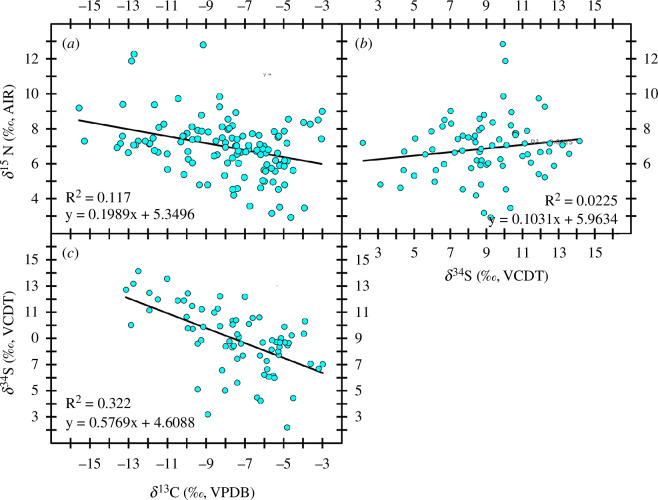
(*a*– *c*) Isotopic compositions of sea turtle bone collagen extracted from whole humeri cross sections (for figures including ST75, see electronic supplementary material, figures S6 and S7).

A large fraction of analyses (99.6% for *δ* ^13^C and *δ* ^15^N and 77.4% for *δ* ^34^S; electronic supplementary material, table S1 and text 6) met standard quality control metrics for evaluating the integrity of archaeological collagen isotopic compositions. Data that did not meet these criteria were removed from further consideration. Results from stable isotope analyses of collagen extracted from whole-bone cross sections reflect an average of diet over the last 10–30 years (based on observations of growth features observed during sampling; see §5 and electronic supplementary material, text 1) of an individual turtle’s life history and are presented in figure 5 and electronic supplementary material, table S1.

Stable carbon isotope compositions show an extremely wide range of variation spanning 19.4‰ (*n* = 122). However, removing one individual (ST75), which produced an extreme outlier *δ* ^13^C value of −22.4‰ (the only *δ* ^13^C outlier identified by a generalized extreme studentized deviate test, *R* = 4.634, *R*_crit_ = 3.451, *p* < 0.05) reduces this range significantly to 12.5‰ (*n* = 121, spanning from −3.0‰ to −15.6‰). Given this large divergence in foraging behaviour and diet (see §4), ST75 was excluded from correlation analyses (for figures including ST75, see electronic supplementary material, figures S6 and S7). Both *δ* ^15^N and *δ* ^34^S also show large ranges of 9.9‰ (*n* = 122, spanning from 2.9‰ to 12.8‰) and 12.0‰ (*n* = 74, spanning from 2.2‰ to 14.2‰). Significant relationships were observed between *δ* ^13^C and *δ* ^15^N (*n* = 121, Pearson’s *r* = −0.394, *p* < 0.001) and *δ* ^13^C and *δ* ^34^S (*n* = 73, Pearson’s *r* = −0.570, *p* < 0.001) but not between *δ* ^15^N and *δ* ^34^S (*n* = 73, Pearson’s *r* = +0.151, *p* = 0.201).

**Figure 6.**
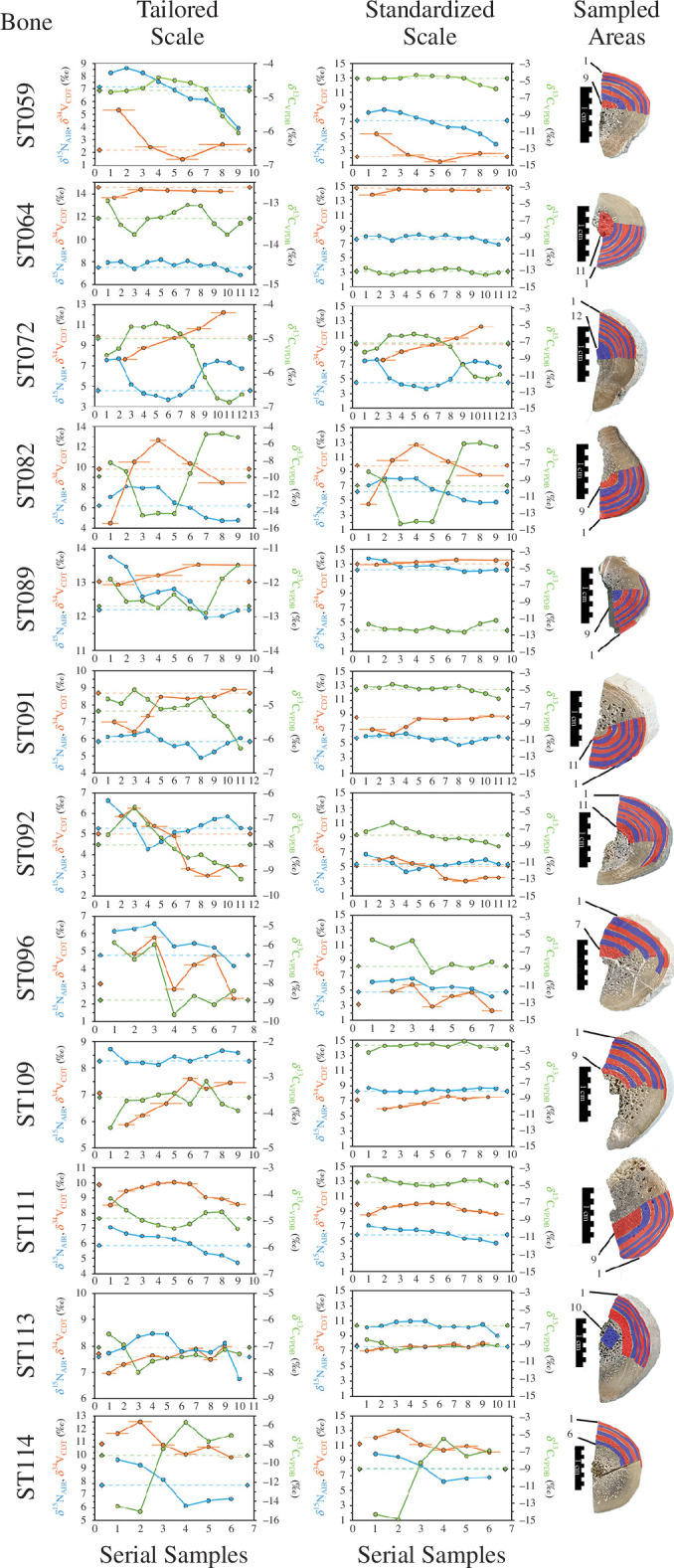
Locations and isotopic compositions of collagen extracted from serially sampled humeri. Dashed lines between diamond symbols show isotopic compositions measured in whole-bone cross sections. Serial sample numbers start from the periosteal (outer; most recent) surface and move inwards. The tailored scale (left column) shows data at scales optimized to observed isotopic variation for each sample. To facilitate inter-sample comparisons, standardized scale (middle column) shows data at the same scale for all samples. The colour alteration between blue and red in sample areas (right column) serves to accentuate transitions between serial sampling (images composed during sample cutting). Notes taken on the number of GLs in each sample are provided in electronic supplementary materials, text 1. While some serial samples correspond to single GLs (and likely represent 1 year of growth) most incorporate more than 1 year’s growth.

Results from serially sampled bone represent shorter annual or multi-annual periods of time and are presented in figure 6 and electronic supplementary material, table S1 (and electronic supplementary material, text 1). While numbers of annual growth layers (GLs) (defined by lines of arrested growth, or LAGs; see §5) could not be systematically counted during serial sampling, observations of numbers of LAG-like features encountered while cutting sample increments (electronic supplementary material, text 1) suggest that serial samples taken from towards the periosteal side of humeri span a timeframe of at least 15–30 years. However, owing to extensive resorption observed towards the endosteal area of nearly all humeri, which obscure LAG visibility, the timeframes represented could be longer. Across all 12 serially sampled specimens, diverse patterning is apparent with intra-individual ranges in *δ* ^13^C, *δ* ^15^N and *δ* ^34^S differing by as much as 8.8‰ (as low as 0.8‰ and high as 9.6‰), 4.2‰ (as low as 0.6‰ and high as 4.7‰) and 7.6‰ (as low as 0.6‰ and high as 8.1‰), respectively.

Ranges and isotopic niche area calculations (sample size corrected standard ellipse areas (SEAc)) for *δ* ^13^C and *δ* ^15^N of all serially sampled individuals, as well as for the wider sample set (i.e. all whole-bone collagen), are provided in electronic supplementary material, table S6, alongside the same metrics for VanderZanden *et al*.’s serially sampled keratin from adult green sea turtles collected in 2009 (*n* = 21, from a nearby nesting beach [43]) and the skin samples from green sea turtles (*n* = 183, sampled from the same region) collected between 2009 and 2011 [42]. The average range and SEAc for serially sampled archaeological bone (*n* = 12, mean SEAc = 2.81 ± 3.26, SEAc ranged from 0.25 to 12.88; Shapiro–Wilk *W* = 0.763, *p* = 0.003) is significantly (Mann–Whitney *U* = 44, *p* = 0.002) larger than that observed among the published keratin (*n* = 21, mean SEAc = 0.50 ± 0.40, SEAc ranged from 0.03 to 1.40; Shapiro–Wilk *W* = 0.904, *p* = 0.042). While bone data incorporates a longer time frame, which could include time periods before a turtle took up residence in the study area (i.e. sample increments from the earliest forming bone; higher increments numbers in figure 6), we note that most dramatic shifts observed in bone serial samples occur (or are at least represented) in the serial sample increments representing the latter stages of life (i.e. lower increment numbers in figure 6). Archaeological whole bone (*n* = 119, SEAc = 13.82, excluding ST75) and published skin sample data (*n* = 183, SEAc = 2.92) from 2009 to 2011 are also divergent, but given the different periods represented by these data (long-term signals from bone versus short-term signals from skin), this difference is difficult to interpret.

**Figure 7.**
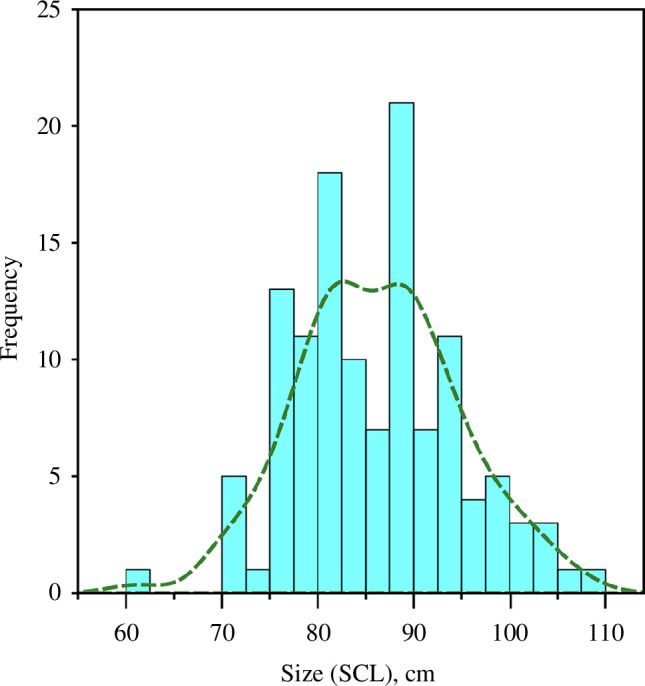
Histogram showing frequency (bars, binned at 2.5 cm intervals) and kernel density (dashed line) of reconstructed SCL based on humeri measurements.

Variations in relative turtle size (straight carapace length, SCL), as estimated through humerus minimum width measurements [53], are reported in electronic supplementary material, table S1. Reconstructed SCL spanned 61.1 to 107.6 cm with a mean of 86.2 cm (*n* = 122; figure 7). Except for ST1, estimated at 61.1 cm, all SCL estimates exceeded 70 cm with most (*n* = 91, 74.6%) being greater than 80 cm. Following Zug *et al*. [53] comparison of SCL and estimated age, the vast majority of green sea turtles included in this study were likely roughly 30 years of age or older. We also draw on the analysis of Eguchi *et al*. [60] of green sea turtles in San Diego Bay to convert SCL estimation to approximate weight in kg (electronic supplementary material, table S1). Excluding ST1 (with an estimated weight of 29.9 kg), reconstructed weights spanned *ca* 43.1 to 192.8 kg with a mean of 89.8 kg. As these weight estimates are based on data that are themselves estimations, we considered them speculative, but we note that they, along with the estimated ages, align well with expected demographics of turtles targeted by Caymanian turtle harvesters at the Miskito Cays for soup production at the Key West Turtle Kraals. We found no significant correlations between estimated SCL (or, by extension, relative approximate age) and *δ* ^15^N (*n* = 121, Pearson’s *r* = −0.069, *p* = 0.449), *δ* ^13^C (*n* = 121, Pearson’s *r* = +0.069, *p* = 0.453) or *δ* ^34^S (*n* = 74, Pearson’s *r* = −0.138, *p* = 0.241) or estimated weight and *δ* ^15^N (*n* = 121, Pearson’s *r* = +0.178, *p* = 0.063), *δ* ^13^C (*n* = 121, Pearson’s *r* = −0.046, *p* = 0.615) or *δ* ^34^S (*n* = 74, Pearson’s *r* = −0.114, *p* = 0.334). As green sea turtles in the region typically reach adulthood between approximately 25 and 35 years of age (and as little as 15 years [61]; for a review, see [62]), when SCL measures approximately 82–117 cm [63], we expect that nearly all of our samples come from turtles that were adults when slaughtered. It is also worth noting that general observations (reported in electronic supplementary material, text 1) of the number of LAG-like features visible (towards humeri periosteal surfaces), as well as the advanced states of resorption present (towards humeri endosteal areas) provide further supporting evidence that most of our sample comes from mature adults (and, at minimum large juveniles), which had attained advanced ages of >30 years.

Based on historical records, it is extremely unlikely that our sample includes specimens from other sea turtle taxa. Species identification based on osteological assessment of humeri morphology (§5) confirms that all specimens come from green sea turtles. Nonetheless, to independently confirm that isotopic variation within our dataset is not a function of taxonomic diversity, we applied ZooMS, a peptide mass fingerprinting technique that can differentiate between all living sea turtle species [64], to a sample of 16 individuals with whole-bone cross-section isotope compositions around the margins of the dataset (electronic supplementary material, figure S8). Although one of the 16 samples lacked one of the peaks reported in the reference spectra published by Harvey *et al*. [64], mass spectra from all samples were consistent with those of green sea turtle (electronic supplementary material, text 2).

## 4. Discussion

Before interpreting isotopic variation in our dataset, it is important to consider the extent to which our sample reflects a population of green sea turtles that were (i) caught in the Miskito Cays and (ii) longer term residents of the Miskito Cays environs as opposed to being migrants from other areas. This can help to contextualize the patterns we have observed as being more or less representative of the local region. With respect to catch location, while records for shipments received by the Key West Turtle Kralls do not survive, it is clear that, particularly by the mid-twentieth century, the cannery’s primary source of green sea turtles was from the Miskito Cays and that the main turtling vessels, particularly the *Adams* (figures 1 and 3), focused their harvesting efforts in that area [5,9]. Therefore, while we cannot say that these samples come exclusively from Miskito Cays harvests, given the dominance of this location in turtling activities at the time, it is unlikely that our sample includes turtles from other areas. We, therefore, consider our sample to reflect a population of turtles harvested in and around the Miskito Cays.

With respect to the likelihood that these turtles were longer term residents of the Miskito Cays, first-hand accounts of the TEK used by Caymanian turtlers, dating to the timeframe of our sample [26,27,30], as well as historical accounts from the Key West Turtle Kraals, provide insight into mobility for turtles in our sample, which can help to constrain interpretations. The techniques used to harvest green sea turtles depended on a detailed knowledge of predictable, cyclical behaviour of turtles that foraged in the area’s seagrass meadows [7,26,27,30,36]. According to accounts of Caymanian turtler TEK, the seagrass meadows and surrounding habitats of the Miskito Cays were home to turtles for extended periods of time during which individuals would repeat a daily cycle of spending nights in or near specific coral hollows nearer the coast, and undertake short daily return migrations of a few miles to nearby turtle grass meadows [7,26,27,30,36]. For a portion of the year, the region also contained green sea turtles that were passing through from more northerly regions on their southward migration to a major breeding aggregation site at Tortuguero, Costa Rica [36] (figure 1). It is therefore possible that Caymanian turtlers could have accessed green sea turtles that fell into either of these residential or migratory groups. However, the TEK used by Caymanian turtlers was specifically oriented towards capturing turtles as they emerged from their nightly sleeping areas associated with specific coral or rock hollows [7]. This means that our sample is most likely composed primarily of turtles that have an established behavioural pattern in the area. Moreover, interviews with veteran turtling captains noted that changing turtle activity associated with the mating season did not impact their rate of success with capturing turtles, aside from a dip observed during periods when fewer turtles were taken, which was attributed to more individuals having migrated away for breeding activities [27].

Other anecdotal evidence supports the strong residential homing sense for green sea turtles inhabiting the region. In a well-documented example, a particularly large turtle (identified by initials carved in its plastron and distinctive flipper markings) was recaptured at the same coral hollow after being previously caught and delivered to Key West [7]. An October hurricane (Tannehill’s No. VII, 1924 [8]) had enabled mass escapes from the Key West Turtle Kraals and allowed this turtle to return to and reclaim its nightly sleeping area where it was caught again by the same turtler shortly thereafter. While interviewing turtling captains in the 1950s, Archie Carr documented numerous instances of this recapture process at a wide range of spatial and temporal scales [7,9]. This strong homing ability and sense of place, coupled with the fact that Caymanian TEK was predicated in part on exploiting these predictable residential behaviours, suggests that green sea turtle populations harvested in our study area were less likely to include individuals that were simply migrating through the area and much more likely to reflect a population that had longer term connections to the Miskito Cays region and its food resources.

In this context, while we expect that sampled turtles had been caught and spent considerable time in the Miskito Cays, we should also evaluate the extent to which the sampled bone (i.e. whole cross-sections of humeri, which reflects longer term averages of diet and mobility) are likely to incorporate isotopic signals from time spent elsewhere (i.e. before turtles took up residence in the Miskito Cays). Based on size estimations (SCL; figure 7) and general age observations of LAG-like features (electronic supplementary material, text 1), these turtles were likely overwhelmingly adults. However, the presence of large juveniles (which will have spent a comparatively larger fraction of their lives moving around, including living offshore) or older individuals that had only recently taken up residency in the area cannot be ruled out. Beyond establishing that the turtles included in this study had likely spent enough time in the Miskito Cays to establish behavioural patterns required to be caught with Caymanian TEK, it is therefore not possible to assess the amount of time (be it months or decades) each turtle had spent in the Miskito Cays before capture. While this issue necessarily remains uncertain for historical samples like ours, we note that no correlation between isotopic composition and reconstructed SCL was observed, suggesting that the patterns we identify below do not reflect ontogenetic diet shifts associated with earlier life stages.

### 4.1. The isotopic ecology Key West Kraal turtles

In the context of isotopic compositions of primary producers (collected in 1976 and 1977) from the Miskito Cays [65], which, in the broader temporal context of historical turtle harvesting in the region, are nearly contemporaneous with our samples, the wide range of *δ* ^13^C values observed among our sample of green sea turtles (figure 5*a,b*) is consistent with grazers feeding on a range of marine algae and seagrasses. The unusually low *δ* ^13^C for ST75 (electronic supplementary material, figures S6 and S7) could be consistent with a diet specializing in terrestrial vegetation, encountered as flotsam, at river mouths or marine primary production with very low *δ* ^13^C values such as red algae.

Given that adult and large juvenile green sea turtles in the Caribbean are thought to be herbivorous (though some sponge consumption has been observed [36]), the large range of *δ* ^15^N observed (figures 5*a* and 4*c*) requires closer examination. Variability in consumer *δ* ^15^N can be related to trophic differences and baseline variations [66,67]. In this case, previous research exploring *δ* ^15^N values of whole tissues versus single amino acids (which provide a proxy for food web baseline *δ* ^15^N) indicates that variability consistent with what we have observed here reflects baseline rather than trophic (i.e. increased omnivory) variation [42]. In that context, a small but significant relationship observed between *δ* ^13^C and *δ* ^15^N suggests that variation in *δ* ^15^N is driven in part by the processes governing variation in *δ* ^13^C. Low *δ* ^15^N has been observed among seagrasses around the globe, reflecting symbiotic relationships that many seagrasses have with N-fixing bacteria in their rhizomes [68–70]. These symbiotic relationships with N-fixing bacteria contribute nitrogen derived from dissolved N_2_, which is typically ^15^N depleted relative to other sources of dissolved inorganic nitrogen (DIN) in marine environments [71]. This correlation fits well with green sea turtles foraging along a spectrum of algae and seagrass.

Stable sulfur isotope compositions (figure 5*b,c*) show a very large range both for a marine consumer and for specimens from a single archaeological deposit. Addressing research question 1 (to what extent can seagrass-intensive diets impact marine consumers *δ* ^34^S?), a majority of data diverge from the high *δ* ^34^S expected from marine fauna [72,73], clearly demonstrating the potential for seagrass use [45–48] to dramatically influence the isotope compositions of slowly remodelling tissues, like bone collagen, in marine consumers. A strong correlation between *δ* ^13^C and *δ* ^34^S suggests that there are two distinct, prevailing dietary sources—one consistent with local seagrasses (higher *δ* ^13^C and lower *δ* ^34^S), and one with other marine foods like algae (lower *δ* ^13^C and high *δ* ^34^S, see below) [47,74].

Interestingly, although we found stronger correlations between *δ* ^13^C and *δ* ^15^N as well as *δ* ^13^C and *δ* ^34^S, no correlation was found between *δ* ^34^S and *δ* ^15^N, indicating that the processes governing variation in these two isotope systems, across the spectrum of green sea turtle diets, are not as strongly linked. Complexities in processes governing *δ* ^15^N variation in marine benthic environments could be masking these relationships. Use of ^15^N-depleted nitrogen sourced from N-fixing bacteria, for instance, is influenced by the availability of other (less energetically costly) sources of DIN [75], which could create heterogenous patterns in primary producers’ and their consumers’ *δ* ^15^N at a wide range of spatiotemporal scales. With respect to baselines, variation in *δ* ^15^N can also arise from differences in both DIN source pools and the ways in which nitrogen is cycled before it is assimilated by aquatic primary production [67,76]. In contrast to *δ* ^13^C and *δ* ^34^S, both of which are in part regulated by cycling that incorporates isotopically homogeneous source pools (dissolved carbon dioxide, bicarbonates and sulfates), source nitrogen pools across the water column can be more variable and nitrogen cycling is often governed by diverse spatial and temporal parameters [67,76]. While not statistically significant, it is interesting to note that the small number of individuals with particularly high *δ* ^15^N (>+9.0‰, *n* = 12) have lower *δ* ^13^C (<−7.0‰) and higher *δ* ^34^S (>+9.0‰), suggesting that their foraging behaviour relied less heavily on seagrass habitats. Although anecdotal in nature, this suggests that isotopic variation arising from differing nitrogen sources and cycling processes may be greater in non-seagrass habitats in this region, where green sea turtles would forage on other foods such as algae or sponges. These data clearly suggest that the processes underlying *δ* ^15^N variation are more intricate than we can disentangle using isotopic compositions from consumers’ whole-bone cross sections. Future research using *δ* ^15^N analyses of individual amino acids may be able to resolve the processes governing the apparent disconnect between how variation in *δ* ^15^N relates to *δ* ^13^C and *δ* ^34^S and could have the potential to advance reconstructions of sea turtle habitat use (e.g. differentiating between preferences for habitats focusing to greater/lesser degrees on areas with different DIN pools).

### 4.2. Archaeological and anthropological relevance

Stable sulfur isotope compositions concretely demonstrate how marine consumers that use seagrass meadow habitats can have what would traditionally be seen as ‘non-marine’ isotopic compositions [72], a finding which has broad implications for the ways in which *δ* ^34^S is typically interpreted in archaeological research. It is important to recognize that this *δ* ^34^S variation is not connected to marginal resources, but, rather, a globally distributed habitat and major source of primary production in coastal regions of six continents, as well as an economically significant food animal. Green sea turtles are easily collected on nesting beaches around the globe and saw extremely broad use among coastal peoples across their range, including around the Atlantic, Pacific and Indian Oceans as well as the Mediterranean Sea, making them a preferred protein source and culinary phenomenon in many societies [19,77]. Moreover, the impact of seagrass consumption on the isotopic composition of economically significant taxa is not limited to green sea turtles. As seagrass-influenced *δ* ^34^S values are passed up and along food webs, so too will fish, bivalves and other marine organisms show this pattern. Indeed, this has been archaeologically observed to smaller degrees in sheepshead (*Archosargus probatocephalus*) in nineteenth-century contexts from New Orleans, Louisiana on the Gulf of Mexico [78] and has been invoked to explain *δ* ^34^S variation in a variety of ecological contexts (e.g. [79–81]). Put simply, the broadest implication is that, where food animals from seagrass-influenced food webs were eaten by humans, human bone collagen *δ* ^34^S values may not provide a robust indicator for marine-intensive archaeological diets.

In this context, it is also worth noting that a common assumption in archaeological [72] (and occasionally ecological; e.g. [82]) interpretations, involving the potential for freshwater sulfur inputs to cause lower *δ* ^34^S in consumers that forage in estuarine systems, is not a realistic explanation for the observation of lower *δ* ^34^S variation in our dataset or in marine food webs more generally. Fry & Chumchal [83] demonstrate, in a large study of fish at different points along an estuarine salinity gradient, that seawater sulfate has a strong influence on the *δ* ^34^S compositions in aquatic food webs even at very low salinities. This makes sense on the basis of simple mass balance, as seawater has sulfate concentrations that are an order of magnitude higher than typical freshwater sources [84]. This means that in most circumstances freshwater sulfate inputs are unlikely to be a primary driver of *δ* ^34^S variation for marine and estuarine ecosystems.

In the context of ethnographic research, our results have other potential culinary implications. Historical and Indigenous sources, from those harvesting sea turtles both around the Miskito Cays [26,27,36] and further abroad [85–88], note that the flavour of green sea turtle products has cultural significance and is strongly linked to the foods turtles eat, with the most desirable green sea turtles coming from particular seagrass pastures, and less desirable turtles coming from locations where primary production is dominated by algae [36,86,88]. Because these differing flavour profiles are associated with turtle diets that should leave a clear isotopic signature, our data may offer a novel way of exploring sensorial dimensions such as personal and collective flavour preferences for sea turtle products in the past. In the context of our data, we can see that long-term diets of turtles had different emphases, with some individuals focusing primarily and consistently on seagrass, and others incorporating more marine algae. We have also observed one individual (ST75) with a diet focusing on foods with extremely low *δ* ^13^C values, such as red algae and/or non-marine vegetation (e.g. specializing in river mouth flotsam; and given this sample’s *δ* ^34^S of +10.0‰, the latter seems more likely). Given the strong aversion we see in firsthand accounts to flavour profiles from green sea turtles associated with non-seagrass habitats, described as creating ‘rank’ [36], ‘stinking’ [86] or ‘musky’ [9], as opposed to ‘sweet’, turtle products, it is perhaps not surprising that this extreme diet is represented by a lone individual.

Based on accounts from peoples involved in historical green sea turtle fisheries around the Miskito Cays, it is likely that the products created from these animals would have had differing flavour profiles. In the context of the soup canneries where these individuals were processed, the integration of calipash and calipee from up to a dozen turtles per day into soup outputs [5] would likely have mitigated against strong flavour variation. Nonetheless, in contexts where people were consuming green sea turtles caught at sea (i.e. not at nesting beaches), TEK could have allowed probabilistic selection of turtles with desired flavour profiles that, as we show here, could be identified isotopically in archaeological turtle bone assemblages. In that context, with further ground-truth testing, perhaps in collaboration with Indigenous Miskito peoples’ culinary knowledge and ongoing turtle harvesting activities, isotopic data from sea turtle remains could shed light both on TEK and culinary preferences in the past.

### 4.3. Ecological and conservation relevance

The strong negative correlation that we have observed between *δ* ^13^C and *δ* ^34^S would be expected for animals grazing primarily on material from the lower portions of seagrass vegetation [23,49–52], and provides an indicator for resolving the importance of this key food in the diets of green sea turtles in the Caribbean. Seagrasses have higher *δ* ^13^C than other marine primary production [65] owing to slower CO_2_ diffusion rates, thus reducing the extent of photosynthetic discrimination against ^13^C, and, owing to an ability of seagrasses to assimilate HCO_3_^−^, contributing comparatively ^13^C-enriched carbon [74]. Foraging in seagrass food webs, therefore, often generates distinctively high *δ* ^13^C profiles in local consumers [65]. However, isotopic research [42] on green sea turtles from the study region, sampled between 2009 and 2011, has been unable to assign variation in turtle *δ* ^13^C values to relative amounts of seagrass consumption owing to natural isotopic variation in these plants. Indeed, a 1982 study [65] of isotopic variation in seagrass, algae and other primary production around the Miskito Cays was one of the first to reveal the potential complexities of interpreting marine energy pathways based on *δ* ^13^C alone, owing to overlapping isotopic compositions between these sources of primary production.

In their still-definitive 1996 review of *δ* ^13^C variation in seagrasses, Hemminga & Mateo [74] highlight the potential for theorizing connections between *δ* ^13^C and *δ* ^34^S to unravel these complexities and better understand seagrass ecosystems, but note that no work had been directed to this end. Despite the growth in stable isotope studies, this continues to be the case, with relatively few published studies engaging with the unique complexities co-driving *δ* ^13^C and *δ* ^34^S variation in seagrass ecosystems. Considering key elements of the known parameters of seagrass *δ* ^13^C and *δ* ^34^S variation in relation to varying spatial and temporal scales, from intra-plant to inter-species and inter-regional contexts, can help resolve seagrass contributions to consumer diets. With respect to *δ* ^34^S variation, it is well documented that sulfide intrusion is strongest near the base of the plant (i.e. lower portions of leaves) [47] and grazers focusing on this portion should therefore have the most sulfide-influenced (i.e. low) *δ* ^34^S values. With respect to *δ* ^13^C variation, it is important to note that while seagrass *δ* ^13^C values do show variation driven by geographical differences in isotopic composition of CO_2_ sources (e.g. being closer to or further from a mangrove or river mouth) [89–93], iridescence, depth [94–97] and productivity [98], they do not show strong systematic intra-plant variation in *δ* ^13^C across a plant structure [89]. One exception to this observation is intra-plant variation that occurs with depth (driven by the influence of iridescence on rates of photosynthesis), but this is unlikely to be relevant in our context both because these isotopic differences are mainly observed under 10 m below the water’s surface (lower than the main seagrass species, turtle grass, in the study region is typically found [95]) and because this does not influence the lower portion of the plants (which are more likely to be fully shaded) favoured by green sea turtles.

Putting these drivers of *δ* ^13^C and *δ* ^34^S variation together, we have a framework where green sea turtles’ preferred portion of seagrass is most likely to have higher *δ* ^13^C and lower *δ* ^34^S values. In contrast, the other major food sources as seen in regional observational and stomach content studies (green algae, Chlorophyceae and red algae, Rhodophyceae, totalling 8.8% on average) [36] will be characterized by comparatively lower *δ* ^13^C and higher *δ* ^34^S values. By linking *δ* ^13^C and *δ* ^34^S, which influence seagrass (and their consumers’) isotope compositions in different, independent ways, we provide a more robust measure showing that, while many turtles focus on seagrass, a substantial fraction (upper left quadrant of figure 5*b*) have long-term diets focused on other foods such as algae. This finding provides an opportunity to address part of research question 2 (how variable are historical sea turtle diets?) as it allows for more detailed interpretation of foraging behaviour than previous isotopic work, based only on *δ* ^13^C and *δ* ^15^N, which suggests a primary focus on seagrass for most turtles. In contrast, the picture we see from combining *δ* ^13^C and *δ* ^34^S shows that many green sea turtles historically harvested around the Miskito Cays have isotope compositions that better fit a substantive dietary focus on algae and with less seagrass emphasis. This revised perspective fits better with a larger, local stomach content study of green sea turtles harvested from the wider area (encompassing cays as far as 100 km south) in 1976–1977 (within 5–30 years of the harvesting of turtles in our sample) suggesting that while in most cases (on average 88%) the dry weight of stomach contents was dominated by seagrasses, there are examples at some locations where turtle diet was composed up to 65% algae (this most extreme of examples comes from four individuals harvested at Set Net Cays) [36].

While still comparatively rare, over the last few years *δ* ^34^S analyses have been increasingly applied among sea turtle research [81,99–105]. Although some researchers have left observed *δ* ^34^S variation uninterpreted, others have identified the utility of *δ* ^34^S for distinguishing between green sea turtle foraging areas. While these studies have advanced our understanding of the value of *δ* ^34^S for marine turtle research, their discussions of variation in *δ* ^34^S either focus on *δ* ^34^S in isolation or, when connected with other isotope systems, leave interpretation to mixing models. In this context, the importance of connecting mutual drivers of patterning both among isotope systems (i.e. *δ* ^13^C and *δ* ^34^S) and between isotope systems and specific green sea turtle foraging behaviour (i.e. a focus on specific plant portions) have remained unexamined. In highlighting the significance of these connections our findings underscore Hemminga & Mateo’s [74] call for work on the underexplored potential of combining perspectives on structured variation across isotopic systems to address deeper questions about marine food webs.

With respect to serial sampling and intra-individual patterning, we saw a surprising amount of variation compared with previous isotopic research characterizing adult green sea turtle behaviour in the region [43]. Our approach to serial sampling humeri (see §5) aimed to include as few annual GLs (as determined by observation of LAG-like features) as possible per increment but many sample increments represent variable amounts of time (with some including five or more GLs; see electronic supplementary material, text 1, for detailed overview). It is also worth bearing in mind that, for serial samples that formed in earlier periods in a turtle’s life, these data could reflect time periods from before the individual took up residence in the Miskito Cays. Furthermore, while we expect that most individuals would have been slaughtered within a year of capture, it is possible that post-capture diets could influence the isotopic composition of the first serial sample increment.

Serial sampling of 12 humeri shows a very wide range of temporal trends in individual dietary behaviour profiles. While many individuals show little or very gradual variation, a quarter (ST72, ST82 and ST114) show dramatic shifts, including both sharp and incremental as well as unidirectional and sinusoidal patterns. Directionality of *δ* ^13^C and *δ* ^34^S shifts generally support interpretations of underlying factors (seagrass as a key driver) we have explored while interpreting data from whole-bone cross sections. In particular, serial sample sequences that show large isotopic shifts are marked by opposing patterns in *δ* ^13^C and *δ* ^34^S variation, although patterns are occasionally somewhat ‘blurred’ owing to serial sample consolation needed to analyse *δ* ^34^S (requiring eight times the material compared with *δ* ^13^C; see §5).

These data offer a valuable comparative context against which to evaluate behaviour seen in more recent populations, providing a framework within which to address the remainder of research question 2 (how do historical sea turtle diets compare with more recent populations?) as well as research question 3 (to what degree do individual turtles shift diet specializations over the last years of life?). A study by Vander Zanden *et al*. [43] examined intra-individual behaviour of turtles at a nearby beach and major green sea turtle nesting ground in 2009 through isotopic analyses of serial samples of adult scute keratin GLs. Compared with this study, Vander Zanden *et al.*’s work included a larger number of individuals (*n* = 21, also adults with a similar, albeit slightly smaller distribution of sizes) and generated time series composed of finer grained sub-annual increments that collectively represented temporal spans ranging from 2.5 to 6.5 years per individual. Our data provide much coarser temporal (year to multi-year) increments but offer longer term (10–30+ years) perspectives. Vander Zanden *et al*.’s analysis of this comparatively recent population exhibited high degrees of both intra-individual isotopic consistency in keratin serial samples and inter-individual isotopic variation, indicating an overall generalist population composed of differently specialized foragers. Significantly, among adults, their study found few examples of large shifts in isotopic composition across serial sample sequences (with average individual-level SEAc of 0.50, *n* = 21). Our data help to confirm the longer term existence of foraging specialist behaviour among green sea turtle adults harvested in the Miskito Cays area, extending Vander Zanden *et al*.’s intra-individual observations of this behaviour from 6.5 to 10+ years. On the other hand, despite our smaller sample size and the fact that our specimens were sourced from a specific foraging ground (rather than a breeding site that serves as an aggregation point for individuals from a larger region), we found more individuals with large-scale isotopic changes (with average individual-level SEAc of 2.81, *n* = 12). That proportionately less variation has been observed in more recent populations points to higher levels of adaptability in past turtle foraging strategies around the Miskito Cays. This shows comparatively less fidelity to a foraging location and/or dietary consistency at foraging sites over longer periods of time.

To better understand these patterns as part of a process of longer term change in human impacts on green sea turtle ecology in the Caribbean Sea, it is important to consider the social, economic and political context driving turtle exploitation in the region. While the Miskito Cays have long been the focus of green sea turtle hunting, commerce developments creating stronger links between Indigenous communities and global markets at the end of the 1960s led to a rapid escalation of hunting pressure [106]. Indigenous communities along the Mosquito Coast have been travelling to the Miskito Cays to harpoon green sea turtles since time immemorial, and Caymanian turtlers have been making regular journeys there to net turtles for at least a century [21,40]. These long-term harvesting patterns were disrupted in the mid- to late 1960s when the Nicaraguan government acted to exclude Caymanian turtlers from legally harvesting green sea turtles around the Miskito Cays [6]. Shortly after, in 1968–1969, foreign-owned turtle companies opened factories in nearby population centres along the Mosquito Coast. These began a programme to boost green sea turtle catches, incentivizing Miskito communities to become more integrated with monetary exchange-based markets, in part, by provisioning them with large numbers of nets and other turtle harvesting infrastructure [6,40,106]. This economic shift away from kin-based redistribution networks and toward market economies, coupled with a dramatically expanded capacity for harvesting centred around new turtle processing facilities, focused on the global export of frozen turtle products, constituted a step change in human–turtle interactions around the Miskito Cays [106]. By 1971, a large increase in harvesting of green sea turtles for these new factory facilities was recorded [40]. Although international export of green sea turtle products ended in 1977 when Nicaragua became a signatory to the Convention on International Trade in Endangered Species of Wild Fauna and Flora, growing domestic demand for turtle products has continued to support elevated levels of recorded green sea turtle harvests from the Miskito Cays [38,39].

Given the developments of the late 1960s and early 1970s, our sample represents a snapshot of green sea turtle behaviour in the final decades of what had been a comparatively stable century-or-more-long period of human–turtle relationships around the Miskito Cays. While it is difficult to quantify the extent to which Miskito and Caymanian turtle harvesting activities were sustainable prior to this 1971 shift, it is well documented that these developments, unfolding by the start of the 1970s, resulted in a substantial increase in turtle harvesting, with impacts on this last remaining stronghold for green sea turtle foraging in the Caribbean Sea [39,40]. In this historical context, the greater degree of variation we see in intra-individual foraging behaviour among green sea turtles from our sample, which were primarily harvested around the Miskito Cays over a 20-year period before this episode, appears to be driven by dynamics of intraspecific competition reflecting higher population densities [107].

It is important to note that we do not interpret our data as showing a difference in the kinds of foods eaten by green sea turtles sampled from before and after the harvesting intensity shifts of the early 1970s (i.e. both published data from skin and keratin samples taken in the 2000s [42,43] and archaeological bone samples show a wide range of isotopic compositions and overall variation; electronic supplementary material, table S6), but rather, our data show a difference in the degree to which individuals specialized in isotopically distinctive foods. In other words, it is not that the overall scope of the diet of green sea turtles has changed, but that the extent to which they engage in selective feeding and resource specialization on an individual level differed between populations before and after the step change in harvesting. While a range of potential explanations for this pattern are possible, we consider a reduction in intraspecific foraging competition resulting from human harvesting pressure to be the most parsimonious explanation. For instance, these comparisons involve different tissues, serially sampled bone and keratin, which (having differing amino acid compositions) may record isotopic signatures in slightly different ways. However, this will not have impacted our interpretations as comparisons of bone and keratin in other taxa with consistent diets show only small and systematic differences (the offset between bone collagen and keratin is +1.4‰ for *δ* ^13^C and +0.9‰ for *δ* ^15^N), particularly considering the enormous scale of isotope variation we have observed in this study [108,109]. It is also possible that other environmental pressures not directly related to humans harvesting could be responsible. For instance, changes in variables such as climate, ocean currents, pollution, marine traffic, disease and food web structure can influence marine animal feeding behaviour. While these cannot be categorically ruled out as potential contributors to the foraging behaviour changes we have documented here, we are not aware of specific events or trends in the local region around the Miskito Cays linked to these or other variables that offer an explanation for a reduction in intraspecific foraging behaviour that is more likely than (or as well evidenced as) harvest-driven population density change (i.e. a large drop in competition for preferred resources). In that context, we believe this pattern reflects a scenario in which expressions of green sea turtle foraging behaviour around the Miskito Cays, at the population level, were altered when a steep climb in harvesting freed up preferred habitat, thereby allowing surviving individuals to avoid changing forging specializations owing to competition.

This finding has important conservation implications. First, this means that as green sea turtle populations rebound, the behaviours that we have observed associated with turtle foraging over the last 50 years (i.e. involving a large degree of long-term individual foraging specialization) could change as individuals adopt more adaptable foraging strategies to deal with higher amounts of intraspecific competition [110,111]. In this context, conservation management strategies would need to consider the potential impacts for future increases in variability in green sea turtle ecological strategies. For instance, to the extent that increasing amounts of shifting foraging specializations would result in individuals using different sections of their foraging areas, a re-emergence of this behavioural pattern in future green sea turtle populations could expose individuals to a wider range of threats over the course of their lives [110–112]. Second, our finding of a higher level of adaptability in past green sea turtle populations has conservation implications in the context of recent synthetic arguments, which have used sea turtle isotopic ecology to weigh in on limitations to restocking strategies [2]. For instance, some have argued that the presence of these ecologically distinct strategies within populations, such as the widely observed intra-specific variation in specialist foraging behaviour, means that sea turtles are not ecologically exchangeable. This implies that conservation-oriented restocking efforts that move individuals from one area to another, even within the same genetic management unit, may be problematic because these individuals would not have the same mix of ecological strategies. However, our findings show a greater degree of adaptability in the behaviour of specialist foragers in the past, which appears to have been density and competition mediated, suggesting that, at least for green sea turtles in this critical area of the Caribbean Sea, this kind of restocking approach might not be problematic. In other words, while we would encourage further work (e.g. with archaeological specimens from other locations) to confirm a more widespread presence of this pattern, our results suggest that future green sea turtles in the area could again exhibit higher degrees of ecological exchangeability than some current perspectives allow.

More broadly, from another perspective, these data provide a baseline for one of the most significant remaining forging areas for green sea turtles and one that is closely connected to one of the world’s largest breeding sites [113]. Understanding the geographical origins and connections between breeding sites and populations using different foraging areas is critical for conservation management and modelling. The seagrass meadows of the Miskito Cays are thought to support a large fraction of the breeding population that use nesting areas in Tortuguero. However, large differences occur between methods for estimating the proportion of this green sea turtle nesting population that use the Miskito Cay’s seagrass beds (e.g. genetic, tagging and satellite tracking [8,35,114]). Vander Zanden *et al*.’s insightful study [42] showed how isotopic analyses can offer an additional, complementary tool but emphasized that more data were needed to render this approach practical, arguing that further refinements were needed to improve resolution for accurately assigning individuals from nesting populations to their respective foraging grounds. In that context, these data not only provide a more robust baseline for this key foraging area but indicate that integrating *δ* ^34^S analyses of sampled turtle tissues from other regions will refine the utility of this approach for assessing foraging behaviour and population connectivity in the greater Caribbean Sea and Gulf of Mexico regions.

While *δ* ^34^S analyses of marine fauna have been comparatively rare, perhaps due in part to a perceived lack of utility for differentiating between major sources of primary production in marine settings, this study highlights the multidisciplinary potential that *δ* ^34^S analyses of marine vertebrates have for the fields of archaeology, palaeontology, ecology and conservation management. In the context of recent technological advancements making *δ* ^34^S more affordable and accessible, our results highlight the value of *δ* ^34^S, specifically when coupled with other isotope systems, for disentangling contributions for key pools of primary production. Moreover, as the first application of *δ* ^34^S to a serial sampling of bone, this study demonstrates the potential that this approach has not only for understanding intra-individual behaviour, but also for assessing the directionality of isotopic relationships used for interpreting inter-individual isotopic variation across consumer populations. This study is also the first to use a large sample of archaeological green sea turtle bones, which are commonly recovered across the globe from coastal archaeological sites near locations where sea turtles have been harvested in the past. In that context, and alongside recent smaller scale studies (with a collective global total analyses of 39 green sea turtle bones [99,115,116]), our findings serve to underscore the value of archaeological green sea turtle bone assemblages as vast and almost untapped biomolecular archives for understanding past sea turtle behaviour. Given that many archaeological specimens date to a time before industrial-scale harvesting began, isotopic perspectives from these past sea turtle populations can offer valuable insights into ecological baseline shifts for conservation managers. In this study, we believe that we have only begun to scratch the surface of this potential.

Finally, it is also worth situating our results within the historical journeys and narratives represented by the turtle remains in our sample, which intersect at several points with Archie Carr *et al*.’s work on the first sea turtle tagging studies at Tortuguero in 1955 and the start of marine turtle conservation in the USA [6]. At that time, Atlantic green sea turtle populations were in a rapid decline towards extirpation but those who could speak most authoritatively on them were the harvesters themselves. Turtle schooner captains like Allie Ebanks, of the Key West Turtle Kraals’ *Adams* (figures 1 and 3), shared their TEK with Carr, helping him to develop theories for the green sea turtles’ reproductive cycle, enabled by what at that time seemed to be fantastical feats of migration and homing [7,9,21]. Carr’s groundbreaking tagging work sought to furnish these turtle schooner captains’ stories with the scientific basis crucial for legally protecting the last vestiges of turtle nesting beaches. It was fitting then that some of the initial tags, which offered the first structured empirical evidence for long-distance green sea turtle movements, were returned by Allie Ebanks himself (see table 6 in [29]). They had been collected on the *Adams* from turtles that were caught around the Miskito Cays and ferried to the Key West Turtle Kraals. Over the next 15 years, Caymanian turtle captains working around the Miskito Cays would continue to return turtle tags for Carr’s research. It seems likely, therefore, that some of the thousands of turtle bones recovered during salvage excavations at the Key West Turtle Kraals, a fraction of which have been incorporated into this study, are from turtles that were part of Carr’s early conservation tagging work. In that context, it is worth reflecting on the fact that these same animals are, for a second time, helping us comprehend green sea turtle ecology and, in that vein, continuing to contribute to Carr’s conservation efforts.

## 5. Material and methods

### 5.1. Experimental design

We provide a detailed explanation of our experimental design in electronic supplementary material, text 3, which offers a review of considerations relating to isotopic analyses of collagen from incrementally forming turtle bone tissues (something which has received little consideration in archaeological literature). Electronic supplementary material, text 4, offers a rationale for why acidification, as a pretreatment step used in the purification of bone collagen extracts (which has been avoided by ecological researchers working with other tissues), is an approach well suited for turtle bone isotopic work, with no expected impacts on the integrity of isotopic data.

### 5.2. Isotopic analyses

Samples of cross sections of humeri, taken from near the centre of the diaphysis (a wedge-shaped slice from the endosteal to periosteal sides), were cut into small cubes (*ca* 2 mm^3^) and then demineralized in a 0.5 M HCl solution (refreshed daily until the reaction was complete). Samples were then rinsed to neutrality in type 1 water (resistivity = 18 MΩ cm). In some cases, where samples appeared to have substantial humic contamination from the burial environment (i.e. presented a dark colouration), subsequent treatment with 0.1 M NaOH in a sonic bath (solution refreshed every 20 min until the reaction was complete) was applied. These were then rinsed again to neutrality in type 1 water. Samples were then refluxed in a 10^–3^ HCl (pH 3) solution at 60°C for 36 h. Samples were then centrifuged (1500 rpm for 15 min), and the solubilized fraction was pipetted to a fresh tube, frozen and lyophilized.

Serial samples were taken from bone immediately adjacent to where whole-bone cross sections were sampled. Approximately 10% of specimens were selected for additional serial sampling work based on two criteria. First, we selected specimens that could be more easily serially sampled based on visual cues. Archaeological bone can take on a slight discolouration (akin to staining) that serves to accentuate natural bone growth features, in this case, LAGs which provided contour lines for defining GLs. Many of our specimens showed clearly marked sequences consistent with LAG structures and selection focused on these because they provide a framework for more accurate serial sampling. Hereafter, we use the term LAG-like features (rather than LAG) in acknowledgement of the fact that, without staining and thin sectioning the samples, some of the features we have counted could overrepresent (in cases where multiple intra-LAG lines of porosity give the appearance of distinct LAGs) or underrepresent the number of LAGs present. In several cases, areas of reproachments (i.e. crowding of LAGs), which can occur in latter forming sections bone from older individuals [1], may not have been visible using this approach (i.e. would require thin sectioning) and would be lumped into a single GL (for more detail, see electronic supplementary material, text 1). Second, we selected samples based on the whole-bone cross-section isotopic compositions with the aim of including individuals with both mixed diets and individuals falling closer to the seagrass- or algae-focused end points of the observed isotopic continuum. Note that with respect to the intra-individual isotopic variability we identified among our samples, individuals with pronounced shifts in sequential isotopic compositions included specimens with whole-bone cross-section isotopic compositions from across this spectrum.

Serial samples were demineralized in a 0.5 M HCl solution as with whole-bone cross sections (endosteal–periosteal wedges cut *ca* 8–10 mm thick), but demineralization took place on an oscillating table (50 rpm) to speed up reaction times (solution refreshed twice daily until the reaction was complete). Sequential samples were then removed by hand (visualized through a large halo-lit magnifying lens) using a scalpel blade and progressing from the periosteal surface inwards. The overall goal in serial sampling was to separate as many GLs as possible. In many cases, GLs had begun to separate along LAG interfaces during demineralization and the associated GL would peel away. In other cases, the cleavage of GLs via scalpel cutting was an intricate process that was guided by and followed the complex three-dimensional architecture of GL structures. Given the variation in thickness observed among GLs, it was not always possible to remove material from a single GL and, in many cases, samples clearly incorporated materials from multiple GLs. Detailed notes on the number of GLs for each serial sample as well as the presence of resorption are provided in electronic supplementary material, text 1. Serial samples were then refluxed and lyophilized following the same procedures as for whole-bone cross sections. Note that while all serial sampling was performed on the same general region of respective humeri, because sampling targeted the sections of bone specimens exhibiting the clearest visual evidence for LAGs (i.e. natural staining needed for defining GLs) the precise locations of sampling varied by specimen. Electronic supplementary material, text 5, offers a rationale for why we have selected the above approach in the context of existing sampling approaches available in the literature as well as ways in which our approach might be improved in the future.

Sub-samples of lyophilized collagen were weighed out into tin capsules for EA-IRMS analysis (0.5 mg for *δ* ^13^C and *δ* ^15^N; 8.0 mg for *δ* ^34^S). In some cases, serial samples did not produce enough collagen extract for *δ* ^34^S analyses. In these cases, collagen from sequential serial samples was combined (see figure 6 and electronic supplementary material, table S1). Replicate analyses were performed on 39% of samples for *δ* ^13^C and *δ* ^15^N and 12% of samples for *δ* ^34^S. Sample isotopic compositions were calibrated using three-point calibration curves. Long-term averages (for check standards) and known (for calibration standards) isotopic compositions for isotopic reference materials are reported in electronic supplementary material, table S2. Averages and standard deviations for calibration standards (electronic supplementary material, table S3), check standards (electronic supplementary material, table S4), and sample replicates (electronic supplementary material, table S5) for all analytical sessions are reported in the electronic supplementary material. For *δ* ^13^C, *δ* ^15^N and *δ* ^34^S, systematic errors (*μ*_(bias)_) were ±0.11‰, ±0.22‰ and ±0.26‰, respectively; random errors (μR_(w)_) were ±0.19‰, ±0.31‰ and ±0.12‰, respectively; and standard uncertainty was ±0.22‰, ±0.38‰ and ±0.28‰, respectively [117]. Collagen quality control was assessed using established criteria including carbon (>13.8%) and nitrogen (>4.0%) elemental concentrations [118] as well as liberal C:N ratio criteria [119]. We also compare elemental concentration (%) and respective *δ* values for carbon, nitrogen and sulfur to assess whether isotopic patterns could be driven by contamination from the burial environment [119] (see electronic supplementary material, text 6, for more details). Statistical comparisons were performed in PAST v. 4.13 [120] and involved Pearson’s *r* tests for assessing the significance of correlations between isotopic compositions as well as generalized extreme studentized deviate test for identifying outliers. For group comparisons, a Shapiro–Wilk test was used to assess the normality of distribution. A Mann–Whitney *U* test was used to compare means for groups that were not normally distributed. We used the SIBER package [121] in R v. 3.6.0 [122], through R Studio’s v. 1.2.1335 [123], to quantify standard bivariate ellipse areas (corrected for sample size, SEAc) to quantify isotopic variation within (i.e. across serial samples of bone or keratin) and between (whole-bone cross section or skin) individuals.

### 5.3. Zooarchaeology

Sea turtle humeri were analysed in the University of New Orleans Archaeology Laboratory and Indiana University Bloomington’s William R. Adams Zooarchaeology Laboratory (WRAZL). Taxonomic identifications were assigned following standard zooarchaeological methods [124] centred on the comparison of unknown archaeological bones to modern reference skeletons from relevant sea turtle species and, where appropriate, using landmarks already identified for differentiating humeri from sea turtle species [125]. Modern reference specimens from WRAZL’s reference collection used in this study are listed in electronic supplementary material, text 7. Morphological analysis indicated that all archaeological turtle remains included in this study derived from green sea turtles. Sea turtle SCL estimation was performed using the regression formula given by Zug *et al*. [53] for the relationship between humerus diameter (narrowest diameter taken from the middle of the humeral shaft distal to the deltopectoral crest) and SCL in green sea turtles. Weight estimation used estimated SCL in the model provided by Eguchi *et al*. [60] describing the relationship of SCL to mass for green sea turtles.

### 5.4. ZooMS

Bone collagen samples (~1–2 mg) were resuspended with 100 µl of 50 mM ammonium bicarbonate and digested with 0.4 µg sequencing grade trypsin (Promega, UK) overnight at 37°C [126]. Digested collagen was then co-crystallized with an equal volume of 10 mg ml^–1^ alpha-cyanohydroxycinnamic acid (Sigma-Aldrich, UK), and left to dry for subsequent peptide mass fingerprint analysis using a Bruker Rapiflex matrix-assisted laser desorption ionization time-of-flight (MALDI-ToF) mass spectrometer collecting 50 000 laser acquisitions over the *m/z* range 700–3700. Resultant spectra were then compared to those of all known sea turtles [64].

On selected samples (ST78 and ST89), LC-MS/MS was carried out to improve understanding of the peaks observed in the ZooMS spectra. The LC separation was performed on a Thermo RSLC system consisting of a NCP3200RS nano pump, WPS3000TPS autosampler and TCC3000RS column oven configured with buffer A as 0.1% formic acid in water and buffer B as 0.1% formic acid in acetonitrile. An injection volume of 2 µl was loaded into the end of a 5 µl loop and reversed flushed onto the analytical column (Waters nanoEase M/Z Peptide CSH C18 Column, 130 Å, 1.7 µm, 75 µm × 250 mm) kept at 35°C at a flow rate of 300 nl min^−1^ for 5 min with an initial pulse of 500 nl min^−1^ for 0.6 min to rapidly re-pressurize the column. The injection valve was set to load before a separation consisting of a multi-stage gradient of 1% B to 5% B over 0.8 min, 5% B to 22% B over 5.5 min, 22% B to 31% B over 1.5 min and 31% B to 80% B over 0.5 min before washing for 4 min at 80% B and dropping down to 1% B in 0.5 min (the complete method time was 25 min). The analytical column was connected to a Thermo Orbitrap Fusion Lumos Tribrid mass spectrometry system via a Thermo nanospray Flex Ion source via a 20 µm ID fused silica capillary. The capillary was connected to a stainless steel emitter with an outer diameter of 150 µm and an inner diameter of 30 µm (Thermo Scientific, ES542) via a butt-to-butt connection in a steel union. The nanospray voltage was set at 2000 V and the ion transfer tube temperature set to 275°C. Data were acquired in a data-dependent manner using 10 scans per cycle, an expected peak width of 6 s and a default charge state of 2. Full MS data were acquired in positive mode over a scan range of 350–1200 Th, with a resolution of 120 000, a custom AGC target of 250%, in automatic injection time mode for a single microscan. Fragmentation data were obtained from signals with a charge state of +2 to +3 and an intensity over 25 000. Fragmentation spectra were acquired with a resolution of 15 000 with a normalized collision energy of 30%, an automated AGC target, first mass of 110 Th and an automated max fill time based on a single microscan. All data were collected in centroid mode. Result (.mgf) files were then searched against a local database that included genome-derived COL1A1 and COL1A2 sequences for *Chelonia mydas* and *Caretta caretta* in both standard (electronic supplementary material, 2 and 3) for ST78 and ST89 respectively, and error-tolerant searches (electronic supplementary material, 4 and 5) for ST78 and ST89, respectively, with trypsin (trypsin/P), allowing for one missed cleavage with error tolerant and two missed-cleavages with standard searches. MS1 tolerance of 0.5 Da, MS2 tolerance of 5 ppm and variable oxidation of proline and lysine for all searches; standard searches also included oxidation of methionine and deamidation of asparagine and glutamine residues.

## Data Availability

All data are included in the electronic supplementary material [127]. All proteomic raw data, composed of .MZXML and .MGF files, are made available on FigShare.

## Footnotes

^1^
Kraals are shallow marine pens (figure 3), widely used across the region, created from wood or concrete pilings spaced at intervals wide enough to allow vegetation (for turtle fodder) to wash in but narrow enough to prevent turtle escape.
